# Bacteria in Nanoparticle Synthesis: Current Status and Future Prospects

**DOI:** 10.1155/2014/359316

**Published:** 2014-10-29

**Authors:** Siavash Iravani

**Affiliations:** Biotechnology Department, Faculty of Pharmacy and Pharmaceutical Sciences, Isfahan University of Medical Sciences, Isfahan 81744-176, Iran

## Abstract

Microbial metal reduction can be a strategy for remediation of metal contaminations and wastes. Bacteria are capable of mobilization and immobilization of metals and in some cases, the bacteria which can reduce metal ions show the ability to precipitate metals at nanometer scale. Biosynthesis of nanoparticles (NPs) using bacteria has emerged as rapidly developing research area in green nanotechnology across the globe with various biological entities being employed in synthesis of NPs constantly forming an impute alternative for conventional chemical and physical methods. Optimization of the processes can result in synthesis of NPs with desired morphologies and controlled sizes, fast and clean. The aim of this review is, therefore, to make a reflection on the current state and future prospects and especially the possibilities and limitations of the above mentioned bio-based technique for industries.

## 1. Introduction 

Nanoscience and nanotechnology has attracted a great interest over the last few years due to its potential impact on many scientific areas such as energy, medicine, pharmaceutical industries, electronics, and space industries. This technology deals with small structures and small-sized materials of dimensions in the range of few nanometers to less than 100 nanometers. Nanoparticles (NPs) show unique and considerably changed chemical, physical, and biological properties compared to bulk of the same chemical composition, due to their high surface-to-volume ratio. NPs exhibit size and shape-dependent properties which are of interest for applications ranging from biosensing and catalysts to optics, antimicrobial activity, computer transistors, electrometers, chemical sensors, and wireless electronic logic and memory schemes. These particles also have many applications in different fields such as medical imaging, nanocomposites, filters, drug delivery, and hyperthermia of tumors [1–4].

An important area of research in nanoscience deals with the synthesis of nanometer-size particles of different morphologies, sizes, and monodispersity [5]. In this regard, there is a growing need to develop reliable, nontoxic, clean, ecofriendly, and green experimental protocols for the synthesis of NPs [6–12]. One of the options to achieve this objective is to use natural processes such as use of enzymes, microbial enzymes, vitamins, polysaccharides, biodegradable polymers, microorganisms, and biological systems for synthesis of NPs. One approach that shows immense potential is based on the biosynthesis of NPs using bacteria (a kind of bottom up approach) [6, 7, 11]. The objects of recent studies tend to provide a controlled and up-scalable process for biosynthesis of monodispersed and highly stableNPs. Thus, a wide number of bacterial species have been used in green nanotechnology to research alternative methods for the synthesis of NPs. Researchers have started to use biomass or cell extracts of bacteria for synthesizing NPs. Bacteria are considered as a potential biofactory for the synthesis of NPs like gold, silver, platinum, palladium, titanium, titanium dioxide, magnetite, cadmium sulphide, and so forth. Some well-known examples of bacteria synthesizing inorganic materials include magnetotactic bacteria and S layer bacteria. Most metal ions are toxic for bacteria, and, therefore, the bioreduction of ions or the formation of water insoluble complexes is a defense mechanism developed by the bacteria to overcome such toxicity [13–16]. In this review, most of the bacteria used in nanoparticle biosynthesis are shown. The aim of this paper is, therefore, to make a reflection on the current state and future prospects and especially the possibilities and limitations of the above mentioned bio-based technique for industries.

## 2. Bacteria in Nanoparticle Synthesis

Bacteriapossess remarkable ability to reduce heavy metal ions and are one of the best candidates for nanoparticle synthesis. For instance, some bacterial species have developed the ability to resort to specific defense mechanisms to quell stresses like toxicity of heavy metal ions or metals. It was observed that some of them could survive and grow even at high metal ion concentrations (e.g.,* Pseudomonas stutzeri* and* Pseudomonas aeruginosa*) [47, 55]. Moreover, Brock and Gustafson [56] reported that* Thiobacillus ferrooxidans, T*.* thiooxidans,* and* Sulfolobus acidocaldarius* were able to reduce ferric ion to the ferrous state when growing on elemental sulfur as an energy source.* T*.* thiooxidans* was able to reduce ferric iron at low pH medium aerobically. The ferrous iron formed was stable to autoxidation and* T*.* thiooxidans *was unable to oxidize ferrous iron, but the bioreduction of ferric iron using* T*.* ferrooxidans* was not aerobic because of the rapid bacterial reoxidation of the ferrous iron in the presence of oxygen [56]. Other biomineralization phenomena, such as the formation of tellurium (Te) in* Escherichia coli *K12 [57], the direct enzymatic reduction of Tc (VII) by resting cells of* Shewanella *(previously* Alteromonas*)* putrefaciens *and* Geobacter metallireducens* (previously known as strain GS-15) [58] and the reduction of selenite to selenium by* Enterobacter cloacae*,* Desulfovibrio desulfuricans, *and* Rhodospirillum rubrum* [26] have been reported, as well. Mullen et al. [14] examined the ability of* Bacillus cereus, B. subtilis, E*.* coli, *and* P*.* aeruginosa *for removing Ag^+^, Cd^2+^, Cu^2+^, and La^3+^ from solution. They found that bacterial cells were capable of binding large quantities of metallic cations. Moreover, some of these bacteria are able to synthesize inorganic materials like the magnetotactic bacteria, which synthesize intracellular magnetite NPs [53]. In this section, most of the bacterial species used in nanoparticle biosynthesis are shown (Table 1).

### 2.1. Silver NPs

Saifuddin et al. [22] have described a novel combinational synthesis approach for green biosynthesis of silver NPs using a combination of culture supernatant of* Bacillus subtilis* and microwave irradiation in water. They reported the extracellular biosynthesis of monodispersed silver NPs (~5–50 nm) using supernatants of* B. subtilis*, but in order to increase the rate of reaction and reduce the aggregation of the produced NPs, they used microwave radiation which might provide uniform heating around the NPs and could assist the digestive ripening of particles with no aggregation [22]. Kalishwaralal et al. [59] reported extracellular synthesis of silver NPs (~40 nm) by bioreduction of aqueous Ag^+^ ions with the culture supernatant of* Bacillus licheniformis*. Moreover, well-dispersed silver nanocrystals (~50 nm) were synthesized using* B. licheniformis* [60]. In case of* Bacillus flexus*, spherical and triangular shaped silver NPs (~12–65 nm) were successfully biosynthesized. The NPs were stable in aqueous solution in five-month period of storage at room temperature in the dark. These NPs showed efficacy on antibacterial property against clinically isolated multidrug resistant (MDR) microorganisms [61]. Wei et al. [62] reported the synthesis of circular and triangular crystalline silver NPs (~14.6 nm) by solar irradiation of cell-free extracts of* Bacillus amyloliquefaciens* and silver nitrate (AgNO_3_). Light intensity, extract concentration, and NaCl addition influenced the synthesis of silver NPs. Under optimized conditions (solar intensity 70,000 lx, extract concentration 3 mg/mL, and NaCl content 2 mM), 98.23 ± 0.06% of the Ag^+^ (1 mM) was reduced to silver NPs within 80 min. Since heat-inactivated extracts also mediated the formation of silver NPs, enzymatic reactions are likely not involved in silver NPs formation. The produced NPs showed antimicrobial activity against* B. subtilis* and* Escherichia coli* in liquid and solid medium [62]. Furthermore,* Bacillus cereus* isolated from the* Garcinia xanthochymus* was used for green biosynthesis of silver NPs (~20–40 nm) [18]. The produced NPs showed antibacterial activity against pathogenic bacteria like* E. coli, Pseudomonas aeruginosa, Salmonella typhi, *and* Klebsiella pneumoniae.*

*Pseudomonas stutzeri* AG259, the silver-resistant bacterial strain, isolated from a silver mine, intracellularly accumulated silver NPs along with some silver sulfide ranging in size from 35 to 46 nm [63]. Larger particles were formed when* P*.* stutzeri* AG259 challenged high concentrations of silver ions during culturing, resulting in intracellular formation of silver NPs ranging in size from a few nm to 200 nm [13, 64].* P*.* stutzeri *AG259 detoxificated silver through its precipitation in the periplasmic space and its bioreduction to elemental silver with a variety of crystal typologies, such as hexagons and equilateral triangles, as well as three different types of particles: elemental crystalline silver, monoclinic silver sulfide acanthite (Ag_2_S), and a further undetermined structure [64]. The periplasmic space limited the thickness of the crystals but not their width, which could be rather large (~100–200 nm) [13].

Cell-free culture supernatants of five psychrophilic bacteria* Phaeocystis antarctica, Pseudomonas proteolytica, Pseudomonas meridiana, Arthrobacter kerguelensis,* and* Arthrobacter gangotriensis* and two mesophilic bacteria* Bacillus indicus* and* Bacillus cecembensis* have been used to biosynthesize silver NPs (~6–13 nm). These NPs were stable for 8 months in the dark. The synthesis and stability of silver NPs appeared to depend on the temperature, pH, or the species of bacteria from which the supernatant was used. It was observed that the* A. kerguelensis* supernatant could not produce silver NPs at the temperature where* P. antarctica* could synthesize silver NPs. Therefore, this study provided important evidence that the factors in the cell-free culture supernatants which facilitated the synthesis of silver NPs varied from bacterial species to species [65].

**Figure 1:**
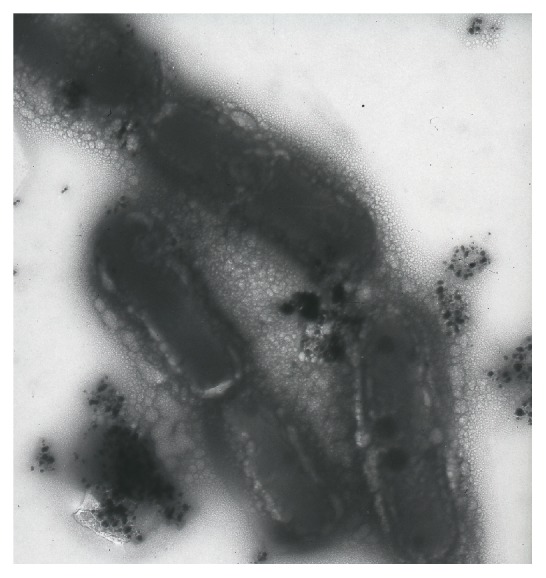
Our research group demonstratedthe bioreductive synthesis of silver NPs using* Lactobacillus casei* subsp.* casei* (an unpublished TEM image recorded from silver NPs synthesized by reaction of silver nitrate solution (1 mM) with* L. casei* subsp.* casei*).

Our research group demonstrated the green biosynthesis of silver NPs using* Lactobacillus casei *subsp.* casei *at room temperature [12] (Figure 1). Previous researchers reported qualitative synthesis of silver NPs by* Lactobacillus *sp., but they did not optimize the reaction mixture. The biosynthesized NPs were almost spherical, single (~25–50 nm), or in aggregates (~100 nm), attached to the surface of biomass or were inside and outside of the cells. The bioreduction of metal ions and stabilization of the silver NPs were confirmed to occur by an enzymatic process. Electron microscopy analysis indicated that the silver NPs were formed on the surface of the cytoplasmic cell membrane, inside the cytoplasm and outside of the cells, possibly due to the bioreduction of the metal ions by enzymes present on the cytoplasmic membrane and within the cytoplasm [12].

A number of metal-reducing bacteria have been isolated and characterized from a variety of habitats, and much work has focused on* Shewanella oneidensis *and* Geobacter *spp. [66]. The biosynthesis of extracellular silver-based single nanocrystallites of well-defined composition and homogeneous morphology utilizing the *γ*-proteobacterium,* S. oneidensis* MR-1, upon incubation with aqueous silver nitrate solution was reported. Further characterization of these particles revealed that the crystals consist of small, reasonably monodispersed spheres in the 2–11 nm size range (~4 ± 1.5 nm) [67].

The rapid biosynthesis of silver NPs using the bioreduction of aqueous Ag^+^ ion by the culture supernatants of* Klebsiella pneumonia*,* E. coli*, and* Enterobacter cloacae* (Enterobacteriaceae) was reported [37]. The synthetic process was quite fast and the produced NPs were formed within 5 min of silver ions coming in contact with the cell filtrate. Piperitone (a natural product which show an inhibitory effect on nitro reduction activity of Enterobacteriaceae) could partially inhibit the bioreduction of silver ions to silver NPs by different strains of Enterobacteriaceae including* K*.* pneumoniae*. As a result of this control experiment, nitroreductase enzymes might be responsible for bioreduction of silver ions. Recently, it was shown that visible-light emission could significantly prompt synthesis of silver NPs by culture supernatants of* K. pneumoniae* [68]. Silver NPs with uniform size and shape (~1–6 nm) were biosynthesized using silver chloride (as the substrate).

When some quantities of NaOH were added to the bioreduction system, silver NPs were successfully prepared with reduction of [Ag(NH_3_)_2_]^+^ by* Aeromonas *sp. SH10 and* Corynebacterium* sp. SH09 [69]. It was speculated that [Ag(NH_3_)_2_]^+^ first reacted with OH^−^ to form Ag_2_O, which was then metabolized independently and reduced to silver NPs by the biomass. The color of the bioreduction system changed from pale yellow to dark yellow. The silver NPs were monodispersed and uniform in size without distinct aggregation and it was observed that the solution containing the NPs remained stable for more than six months. The color change indicated the formation of silver NPs in the reaction mixture, as it is well-known that silver NPs exhibit striking colors (light yellow to brown) due to excitation of surface plasmon vibrations (SPV) in the particles [70].

### 2.2. Gold NPs

It was reported that* Bacillus subtilis* 168 was able to reduce Au^3+^ ions to octahedral gold NPs (~5–25 nm) within bacterial cells by incubation of the cells with gold chloride under ambient temperature and pressure conditions [20, 21]. The reduction processes of chloroaurate and silver ions by* B. subtilis* were found to be different [71]. Gold NPs were biosynthesized both intracellularly and extracellularly, while silver NPs were exclusively formed extracellularly. The gold NPs were formed after one day of addition of chloroaurate ions, while the silver NPs were formed after seven days. Transmission electron microscopy (TEM) micrographs depicted the formation of gold NPs intracellularly and extracellularly, which had an average size of 7.6 ± 1.8 and 7.3 ± 2.3 nm, respectively, while silver NPs were exclusively formed extracellularly, with an average size of 6.1 ± 1.6 nm. The bacterial proteins were analyzed by sodium dodecyl sulfonate-polyacrylamide electrophoresis (SDS-PAGE) before and after the addition of metal ion solutions. Satyanarayana et al. reported that proteins of a molecular weight between 25 and 66 kDa could be responsible for chloroaurate ions reduction, while the formation of silver NPs could be attributed to proteins of a molecular weight between 66 and 116 kDa. They reported that the NPs were stabilized by the surface-active molecules, that is, surfactin or other biomolecules released into the solution by* B. subtilis*.

*Bacillus megaterium* D01 have shown the strong potential of Au^3+^ adsorption [19]. When* B*.* megaterium *D01 biomass was exposed to the aqueous solution of HAuCl_4_, monodispersed spherical gold NPs capped with self-assembled monolayer (SAM) of thiol of 1.9 ± 0.8 nm sizes have been synthesized, extracellularly. The gold NPs were stable without any aggregation over a period of several weeks. Therefore, by addition of dodecanethiol (as the capping ligand) into the reaction mixture monodispersed spherical gold NPs were produced. Moreover,* Lactobacillus *strains, when exposed to gold ions, resulted in formation of gold NPs within the bacterial cells [38]. It was reported that exposure of lactic acid bacteria present in the whey of buttermilk to mixtures of gold ions could be used to grow gold NPs. The nucleation of gold NPs occurred on the cell surface through sugars and enzymes in the cell wall, and then the metal nuclei were transported into the cell where they aggregated to larger-sized particles.

All members of the genus* Shewanella* reported so far are facultatively anaerobic, gram-negative, motile by polar flagella, rod-like, and generally associated with aquatic or marine habitats [72–79]. Most of* Shewanella *species are mesophilic, psychrotolerant, and psychrophilic bacteria [72, 80].* Shewanella alga is* a gram-negative bacillus, which is widely distributed in the environment, and its natural habitats are water and soil [81]. Konishi et al. [82] reported microbial deposition of gold NPs using* S*.* algae*. This bacteriumcan grow anaerobically in medium with lactate or H_2_ as the electron donor and Fe (III) citrate as the electron acceptor. They demonstrated that resting cells of* S*.* algae *were capable of reducing AuCl_4_^−^ ions (1 mM) into elemental gold within 30 min at 25°C over the pH range from 2.0 to 7.0, when H_2_ gas was provided as the electron donor [83]. At pH 7.0, biogenic gold NPs (~10–20 nm) were deposited in the periplasmic space of* S. algae *cells. When the solution pH decreased to below 2.8, some gold NPs were deposited extracellularly. At this pH, the biogenic gold NPs (~15–200 nm) on the bacterial cells exhibited various morphologies. At a solution of pH 2.0, biogenic gold NPs (~20 nm) were deposited on the bacterial cells, and larger gold particles (~350 nm) were deposited extracellularly. Thus, it could be concluded that the solution pH is an important factor in controlling the morphology of biogenic gold NPs and in the location of gold deposition [82]. They observed that the decrease in the soluble Au (III) concentration was presumably caused by its rapid reduction into insoluble gold. In the absence of H_2_ gas, however,* S*.* algae *cells were not able to reduce Au (III) with lactate as an alternative electron donor. Moreover, in a sterile control medium without* S*.* algae *cells, Au (III) was not chemically reduced by H_2_ gas. Thus, resting cells of* S*.* algae *were able to reduce the soluble Au (III) into insoluble gold in the presence of molecular H_2_ as the electron donor [28, 84].

*Escherichia coli *DH5*α* can be used in synthesizing gold NPs (~25 ± 8 nm) [33]. Shapes and sizes of the NPs were not homogenous. They were mostly spherical and a few amounts of triangles and quasi-hexagons were observed, as well. TEM micrographs showed that gold NPs were bound on the surface of the bacteria. Using* E*.* coli*-gold NPs composite, Liangwei et al. made an Hb-coli-nAu-Glassy Carbon electrode, which could be used to achieve the direct electrochemistry of hemoglobin. In another study, biorecovery of gold from jewellery wastes was obtained using* E*.* coli* MC4100 (nonpathogenic strain) and* Desulfovibrio desulfuricans *ATCC 29577 [34]. When these bacteria were exposed to HAuCl_4_ solution (2 mM), gold NPs was formed. In order to control size and shape of the NPs, Deplanche et al. investigated the influence of pH on Au (III) bioreduction using* E*.* coli* and* D*.* desulfuricans*. At acidic pH, spherical gold NPs (less than 10 nm diameter) were produced, but at pH 7.0 and 9.0, a mixture of smaller (~10 nm) and bigger (~50 nm) well-defined triangles, hexagons, and rods were formed.

*Rhodopseudomonas capsulata* showed the ability to produce gold NPs in different sizes, and the shape of gold NPs was controlled by pH [15].* R*.* capsulate* was capable of producing gold NPs extracellularly and the gold NPs were quite stable in the solution. The aqueous chloroaurate ions were reduced during exposure to the biomass and the color of the reaction solution turned from pale yellow to purple. When the pH was adjusted to 4, a number of nanoplates were observed in addition to the spherical gold NPs in the reaction solution. Size and morphology of gold NPs might be affected by AuCl_4_^−^ ions concentration. At lower concentration of AuCl_4_^−^ ions as the substrate, spherical gold NPs (~10–20 nm) were synthesized exclusively, while at higher concentrations, networked gold nanowires were produced in the aqueous solution [49]. The AuCl_4_^−^ ions could bind to the biomass through the main groups of secreted enzymes. These enzymes showed an important role in reduction of AuCl_4_^−^ ions. Bioreduction of Au (3+) to Au (0) and formation of gold NPs might be due to NADH dependent enzymes which were secreted by* R*.* capsulate*. The mechanism of reduction seemed to be initiated by electron transfer from NADH by NADH dependent reductase as the electron carrier.

Lengke et al. [42] controlled morphology of gold NPs using filamentous cyanobacteria, such as* Plectonema boryanum* UTEX 485. They produced cubic gold NPs (<10–25 nm) and octahedral gold platelets (~1–10 *μ*m) interacting* P*.* boryanum *UTEX 485 with aqueous Au (S_2_O_3_)_2_^3−^ and AuCl_4_^−^ solutions at 25–100°C for up to 1 month and at 200°C for one day. The interaction of cyanobacteria with aqueous Au (S_2_O_3_)_2_^3−^ promoted the precipitation of cubic gold NPs at membrane vesicles and admixed with gold sulfide within the cells and encrusted on the cyanobacteria, whereas reaction with AuCl_4_^−^ resulted in the precipitation of octahedral gold platelets in solutions and NPs of gold (<10 nm) within bacterial cells. Moreover, the mechanisms of gold bioaccumulation by cyanobacteria from gold (III)-chloride solutions have shown that the interaction of cyanobacteria with aqueous gold (III)-chloride initially promoted the precipitation of NPs of amorphous gold (I)-sulfide at the cell walls and finally deposited metallic gold in the form of octahedral (III) platelets (~10 nm–6 *μ*m) near cell surfaces and in solutions [43].

Plant-growth-promoting bacteria isolated from Philippine soils were screened for their ability to extracellularly synthesize gold NPs. Extracellular synthesis of gold NPs (~10–100 nm) was determined by incubation of the plant-growth-promoting culture supernatant with gold (III) chloride trihydrate (HAuCl_4_•3H_2_O) for 7 days at 28°C. It was suggested that nitrate reductase was one of the enzymes responsible in the bioreduction of ionic gold [85].

### 2.3. Magnetite NPs

*Desulfovibrio magneticus* strain RS-1 is an anaerobic sulfate-reducing bacterium which also respires and grows with fumarate as the terminal electron acceptor [86].* D*.* magneticus *strain RS-1 accumulated magnetite NPs intracellularly. Most magnetite crystals in the cells were only slightly larger than 30 nm (super paramagnetic NPs) [29]. Klaus-Joerger reported that metals including Co, Cr, and Ni might be substituted into magnetite crystals biosynthesized in the thermophilic iron-reducing bacterium* Thermoanaerobacter ethanolicus* (TOR-39) [13, 54, 87]. This procedure led to formation of octahedral-shaped magnetite (Fe_3_O_4_) NPs (<12 nm) in large quantities that coexisted with a poorly crystalline magnetite phase near the surface of the cells [87]. A more fundamental investigation in the assembly of single-domain magnetite particles into folded-chain and flux-closure ring morphologies by harvested magnetotactic bacterium,* Magnetospirillum magnetotacticum*, was carried out by Philipse and Maas [40]. Magnetic NPs were also assembled into ordered structures when the motion of* M*.* magnetotacticum* (MS-1) was controlled by applying a magnetic field [88].

Fe (III) is an important oxidant of natural and contaminant organic compound in surface and subsurface aquatic sediments [89, 90]. Fe (III) and Mn (IV) could influence the inorganic geochemistry of sedimentary environment by greatly increasing the dissolved concentration of iron, trace metal, manganese, and phosphate [91, 92]. Sulfate-reducing bacteria were capable of producing magnetic iron sulfide (FeS) NPs. Adsorption of radioactive metals by these magnetic iron sulfide NPs occurred due to high surface area (400–500 m^2^/g) which could provide a suitable matrix for the long-term safe storage of hazardous radioactive pertechnetate ion [93, 94]. GS-15 (an obligately anaerobic, gram-negative rod) oxidized simple organic compounds such as acetate, butyrate, and ethanol to carbon dioxide with Fe (III) or Mn (IV) as the sole electron acceptor [53, 95]. Iron-reducing microorganism, GS-15, produced copious quantities of ultrafine-grained magnetite, with size range of 10 to 50 nm, under anaerobic conditions by coupling the organic matter to the reduction of ferric iron [53]. In this process, the nonmagnetic brown amorphic ferric oxide was converted to a black solid material which was strongly attracted to a magnet. But GS-15 was not magnetotactic because the crystals were clearly external to the cells and were not aligned in chains. Lovely et al. [96] investigated the ability of* Alteromonas putrefaciens* to couple the oxidation of potential electron donors (such as lactate, pyruvate, hydrogen, and formate) to the reduction of Fe (III) and Mn (IV). Also they reported that* Pelobacter acetylenicus *and* P*.* venetianus* were able to reduce Fe (III). They demonstrated that* P*.* carbinolicus* was capable of conversing energy to support growth from Fe (III) respiration as it also grew with H_2_ or formate as the electron donor and Fe (III) as the electron acceptor.* P*.* carbinolicus *grew with ethanol (ethanol was metabolized to acetate) as the sole electron donor and Fe (III) as the sole electron acceptor. Growth was also possible on Fe (III) with the oxidation of propanol to propionate or butanol to butyrate if acetate was provided as a carbon source [97].

Jahn et al. [98] discovered a novel mechanism for electron transfer from iron-reducing microorganisms to insoluble iron phases. They monitored iron reduction kinetics with soluble electron acceptors such as ferric citrate, ferrihydrite colloids, and solid ferrihydrite. Roden and Lovley [99] have demonstrated that the marine strain of* Desulfuromonas acetoxidans *was capable of dissimilatory Fe (III) and Mn (IV) reduction. They reported that washed cell suspensions of the type strain of* D*.* acetoxidans* reduced soluble Fe (III)-citrate and Fe (III) complexed with nitriloacetic acid. Ethanol, propanol, pyruvate, and butanol served as electron donors for Fe (III) reduction, as well. In addition, Kashefi and Lovley [100] reported that* P*.* islandicum* owns the ability to reduce Fe (III) at 100°C in a medium with hydrogen as the electron donor and Fe (III)-citrate as the electron acceptor. Cell suspensions of* P*.* islandicum* reduced the following metals with hydrogen as the electron donor: U (VI), Tc (VII), Cr (VI), Co (III), and Mn (IV). The reduction of these metals was dependent upon the presence of cells and hydrogen. In contrast,* P*.* islandicum* could not reduce As (V) or Se (VII). Reducing varieties of metals by* P*.* islandicum* plays an important role in geological phenomena and have application for remediation of metal contaminated water.* Thermus *specieswere able to reduce U (VI), Cr (VI), and Co (III) at 60°C [101]. Thermophilic (45 to 75°C) bacteria showed the ability to reduce amorphous Fe (III)-oxyhydroxide to magnetic iron oxides [102]. They could reduce Cr (VI) and Co (III) at temperatures of up to 65°C, as well [103]. Hyperthermophilic metal reducing microorganisms could help preventing migration of these contaminants by reducing them to less mobile forms. Hot radioactive or metal-containing industrial wastes could potentially be treated in bioreactors containing microorganisms with a metabolism like that of* P*.* islandicum* or their enzymes [100].

Many mesophilic microorganisms which own the ability to use Fe (III) as a terminal electron acceptor could also reduce a variety of metals and metalloids other than Fe (III) [104, 105]. Fe (III)-reducing bacteria and archaea were capable of precipitating gold by reducing Au (III) to Au (0) [28]. The reaction seemed to be enzymatically catalyzed which were dependent on temperature and the presence of hydrogen (as a specific electron donor). Many Fe (III)-reducing microorganisms could reduce forms of oxidized metals, including radio nuclides such as uranium (VI) [89, 95–101] and technetium (VII) [28, 89, 98–103] and trace metals including arsenic (V) [30, 106], chromium (VI) [89, 92, 94, 98, 104, 105], cobalt (III) [1, 15, 30, 89, 92, 106], manganese (IV) [100, 107], and selenium (VI) [82, 108]. Many of these metals and metalloids are environmental contaminants. Therefore, Fe (III)-reducing microorganisms could be used for removal of contaminant metals from waters and waste streams and immobilization of metals in subsurface environments. Microbial reduction of some of these metals may also play an important role in the formation of metal deposits, which may be especially important in hot environments containing metal-rich waters [33, 107–111]. Common product of bacterial iron reduction nanosized magnetic particles, enabled early disease detection and accurate prognosis, and personalized treatment, monitoring efficacy of a prescribed therapy, or study of cellular interaction in a certain biological environment [109, 110]. These particles might have different applications in radionuclide therapy, drug delivery, magnetic resonance imaging (MRI), diagnostics, immunoassays, molecular biology, DNA and RNA purification, cell separation and purification, cell adhesion research, hyperthermia [111, 112], and magnetic ferrofluids for magnetocaloric pumps [113, 114].

### 2.4. Palladium and Platinum NPs

The sulfate-reducing bacterium,* Desulfovibrio desulfuricans *[27, 115], and metal ion-reducing bacterium,* Shewanella oneidensis, *were capable of reducing soluble palladium (II) into insoluble palladium (0) with formate, lactate, pyruvate, or H_2_ as the electron donor [116]. Konishi et al. [52] demonstrated that resting cells of* S*.* algae *were able to deposit platinum NPs by reducing PtCl_6_^2−^ ions within 60 min at pH 7 and 25°C. Biogenic platinum NPs of about 5 nm were located in the periplasmic space. In this case, the cell suspension changed the color from pale yellow to black in 10 min. The black appearance provided a convenient visible signature for the microbial formation of metallic platinum NPs. The observed decrease in aqueous platinum concentration was presumably caused by the rapid reduction of PtCl_6_^2−^ ions into insoluble platinum. In the absence of lactate, however,* S*.* algae *cells were not able to reduce the PtCl_6_^2−^ ions. They reported that the PtCl_6_^2−^ ions were not chemically reduced by lactate. Yong et al. [117] also reported that the sulfate-reducing bacterium* D*.* desulfuricans *was able to adsorb only 12% of platinum (IV) ions on the bacterial cells from 2 mM platinum chloride solution. In another study, Gram-negative cyanobacterium,* P. boryanum* UTEX 485, extracellularly produced Pt (II)-organics and metallic platinum NPs at 25–100°C for up to 28 days and 180°C for 1 day with different morphologies of spherical, bead-like chains and dendritic in the size range of 30 nm–0.3 *μ*m [118].

### 2.5. Selenium and Tellurium NPs

Selenium has photo-optical and semiconducting properties that have applications in photocopiers and microelectronic circuit devices.* Stenotrophomonas maltophilia* SELTE02 showed promising transformation of selenite (SeO_3_^−2^) to elemental selenium (Se^0^) accumulating selenium granules either in the cell cytoplasm or in the extracellular space. In addition,* Enterobacter cloacae* SLD1a-1,* Rhodospirillum rubrum,* and* Desulfovibrio desulfuricans* have also been found to bioreduce selenite to selenium both inside and outside the cell with various morphologies like spherical, fibrillar, and granular structure or with small atomic aggregates.* E. coli* also deposited elemental selenium both in periplasmic space and cytoplasm, and* P. stutzeri* also aerobically reduced selenite to elemental selenium [119]. Under aerobic conditions, Hunter and Manter [120] reported that* Tetrathiobacter kashmirensis* bioreduced selenite to elemental red selenium. A 90-kDa protein present in the cell-free extract was believed to be responsible for this bioreduction. Moreover, Yadav et al. [121] showed that* P. aeruginosa* SNT1 biosynthesized nanostructured selenium by biotransforming selenium oxyanions to spherical amorphous allotropic elemental red selenium both intracellularly and extracellularly. In addition,* Sulfurospirillum barnesii*,* Bacillus selenitireducens, *and* Selenihalanaerobacter shriftii* synthesized extracellularly stable uniform nanospheres (~300 nm) of elemental selenium Se^0^ with monoclinic crystalline structures [122]. Microbial synthesis of elemental selenium (Se^0^) nanospheres resulted in unique, complex, compacted nanostructured arrangements of Se atoms. These arrangements resulted due to the dissimilatory reductions that were subtly different in different microbes. In another study, stable, predominantly monodispersed, and spherical selenium NPs (with an average size of 21 nm) were synthesized using the bacterial isolate* Pseudomonas aeruginosa* strain JS-11. The bacteria exhibited significant tolerance to selenite (SeO_3_^2−^) up to 100 mM concentration with an EC_50_ value of 140 mM. The culture supernatant contained the potential of reducing soluble and colorless SeO_3_^2−^ to insoluble red elemental selenium (Se^0^) at 37°C. It was suggested that the metabolite phenazine-1-carboxylic acid released by strain JS-11 in culture supernatant along with the known redox agents like NADH and NADH dependent reductases was responsible for biomimetic reduction of SeO_3_^2−^ to Se^0^ nanospheres. The authors elucidated that the red colored Se^0^ nanospheres may serve as a biosensor for nanotoxicity assessment, contemplating the inhibition of SeO_3_^2−^ bioreduction process in NPs treated bacterial cell culture supernatant, as a toxicity end point. In other words, the formation of red Se^0^ from SeO_3_^2−^ could serve as a molecular marker, whereas the inhibition of critical bioreduction step was considered as a toxicity end point for the qualitative and quantitative toxicity assessment [123].

Tellurium (Te) has been reduced from tellurite to elemental tellurium by two anaerobic bacteria,* Bacillus selenitireducens* and* Sulfurospirillum barnesii*.* B. selenitireducens* initially formed nanorods of 10 nm in diameter and 200 nm in length were clustered together to form larger rosettes of ~1000 nm but with* S. barnesii* small irregularly shaped extracellular nanospheres of diameter <50 nm were formed [124]. In another study, tellurium-transforming* Bacillus* sp. BZ isolated from the Caspian Sea in northern Iran was used for the intracellular biosynthesis of elemental tellurium NPs. The biogenic NPs were released by liquid nitrogen and purified by an n-octyl alcohol water extraction system. TEM analysis showed rod-shaped NPs with dimensions of about 20 nm × 180 nm. The produced NPs had a hexagonal crystal structure [125].

### 2.6. Zinc Oxide NPs

Zinc oxide (ZnO) NPs have unique optical and electrical properties, and as a wide band gap semiconductor, they have found more uses in biosensors, nanoelectronics, and solar cells. These NPs are being used in the cosmetic and sunscreen industry due to their transparency and ability to reflect, scatter, and absorb UV radiation and as food additives. Furthermore, zinc oxide NPs are also being considered for use in next-generation biological applications including antimicrobial agents, drug delivery, and bioimaging probes [126]. A low-cost and simple procedure for synthesis of zinc oxide NPs using reproducible bacterium,* Aeromonas hydrophila,* was reported. X-ray diffraction (XRD) confirmed the crystalline nature of the NPs, and atomic force microscopy (AFM) showed the morphology of the nanoparticle to be spherical, oval with an average size of 57.72 nm. The antibacterial and antifungal activity was ended with corresponding well diffusion and minimum inhibitory concentration. The maximum zone of inhibition was observed in the ZnO NPs (25 *μ*g/mL) against* Pseudomonas aeruginosa* (~22 ± 1.8 mm) and* Aspergillus flavus* (~19 ± 1.0 mm) [126].

### 2.7. Titanium and Titanium Dioxide NPs

Spherical titanium (Ti) NPs (~40–60 nm) were produced extracellularly using the culture filtrate of* Lactobacillus* sp. at room temperature [39]. These NPs were lighter in weight and have high resistance to corrosion and have enormous applications in automobiles, missiles, airplanes, submarines, cathode ray tubes, and desalting plants and have promising future role in cancer chemotherapy and gene delivery. TiO_2_ (titanium dioxide) NPs has been explored in various biomedical applications such as wound dressing, biosensing, contrast agents, targeted drug delivery agents, antiwrinkle, and antimicrobial and antiparasitic agents owing to their nontoxic and biocompatible properties [127]. These NPs were biologically synthesized by using* Bacillus subtilis*. The morphological characteristics were found to be spherical, oval in shape, individual NPs as well as a few aggregates having the size of 66–77 nm. The XRD shows the crystallographic plane of anatase of titanium dioxide NPs, indicating that NPs structure dominantly corresponds to anatase crystalline titanium dioxide [128]. In another study, biosynthesis of titanium dioxide NPs by a metal resistant bacterium isolated from the coal fly ash effluent was reported. The bacterial strain on the basis of 16S rDNA technique and the biochemical parameters was identified to be* Propionibacterium jensenii* [KC545833], a probiotic, high G + C rich, pleomorphic rod shaped gram-positive bacteria. The produced NPs were smooth and spherical in shape and ranged in size from 15 to 80 nm [127].

### 2.8. Cadmium Sulphide NPs

Several investigations have shown that cadmium sulphide particles could be microbially produced in* Klebsiella aerogenes* [36] and the yeasts such as* Candida glabrata *and* Schizosaccharomyces pombe* [129, 130]. Holmes et al. [36] have demonstrated that the exposure of the bacterium,* K*.* aerogenes*, to Cd^2+^ ions resulted in the intracellular formation of CdS NPs in the size range of 20–200 nm. They also showed that the buffer composition of the growth medium plays an important role in formation of cadmium sulfide crystallites. Cadmium sulphide uptake by* K*.* aerogenes* cells grown in the presence of 2 mM Cd (NO_3_)_2_ could be up to approximately 20% of the total biomass [36].

Synthesis of semiconductor NPs such as CdS, ZnS, and PbS for application as quantum-dot fluorescent biomarkers and cell labeling agents was reported [131]. These luminescent quantum dots are emerging as a new class of materials for biological detection and cell imaging, based on the conjugation of semiconducting quantum dots and biorecognition molecules. Bacteria have been used with considerable success in the synthesis of CdS NPs [33, 85, 94].* Clostridium thermoaceticum *(an acetogenic bacterium) showed the ability to grow autotrophically on CO_2_ and H_2_, utilizing the so-called Wood-Ljungdahl pathway to synthesize acetyl coenzyme A (acetyl-CoA), a starting material for the anabolic processes of these cells [132]. Cunningham et al. [23] reported that* C*.* thermoaceticum* was able to precipitate cadmium extracellularly at an initial concentration of 1 mM. This process was energy dependent and required cysteine. The yellow precipitate of CdS appeared approximately 12 h after the addition of CdCl_2_ and complete removal of the metal from the growth medium was accomplished within 72 h. CdS was precipitated from CdCl_2_ in the presence of cysteine hydrochloride by* C*.* thermoaceticum* at the cell surface and in the medium [23].* Rhodopseudomonas palustris *was capable of producing cadmium sulfide NPs when it was incubated with 1 mM CdSO_4_ at 30°C for 72 h [50]. The researchers of this study found that C-S-lyase (an intracellular enzyme located in the cytoplasm) was responsible for the synthesis of NPs. One of the interesting results of this study was that* R. palustris* transported CdS NPs out of the cell.

In another case,* E*.* coli*, when incubated with cadmium chloride (1 mM) and sodium sulfide (1 mM), showed the capacity to synthesize intracellular semiconductor cadmium sulfide (CdS) nanocrystals [30]. The nanocrystals were composed of a wurtzite crystal phase with a size distribution of 2–5 nm. Nanocrystals formation varied dramatically depending on the growth phase of the cells. Scanning transmission electron microscopy (STEM) and high resolution transmission electron microscopy (HRTEM) revealed that nanocrystals formation increased about 20-fold in* E*.* coli* grown in the stationary phase cells compared to late logarithmic phase cultures.

### 2.9. Zinc Sulfide NPs

Labrenz et al. [25] reported that spherical aggregates of 2 to 5 nm diameter sphalerite zinc sulfide (ZnS) particles were formed within natural biofilms dominated by sulfate-reducing bacteria of the family of Desulfobacteraceae. A combination of geochemical and microbial processes led to ZnS biomineralization in a complex natural system. It is appropriate to mention that the concentration of Zn was significantly reduced to below-acceptable levels for drinking water with the use of this method.* Rhodobacter sphaeroides* has been developed for the synthesis of ZnS NPs with an average diameter of 8 nm [48]. In another study, ZnS NPs (~10.5 ± 0.15 nm) were produced by using* R. sphaeroides* [133].

## 3. Mechanistic Aspects

The ability of bacteria to survive and grow in stressful situations might be due to specific mechanisms of resistance which include efflux pumps, metal efflux systems, inactivation and complexation of metals, impermeability to metals and the lack of specific metal transport systems, alteration of solubility and toxicity by changes in the redox state of the metal ions, extracellular precipitation of metals, and volatilization of toxic metals by enzymatic reactions [134, 135]. For example,* Pseudomonas stutzeri *AG 259 isolated from silver mines has been shown to produce silver NPs [136]. There are several examples of microorganisms-metal interactions which are important in biotechnological applications, including the fields of biomineralization, bioremediation, bioleaching, and microbial-influenced corrosion (MIC) processes. Understanding of MIC processes in terms of microbially mediated localized changes in the surface chemistry of carbon steel, stainless steel, copper alloys, or other ones is gaining growing attention [137]. Bacteria also intervene in mineral precipitation reactions directly as catalysts of aqueous chemical reactions and indirectly as geochemically reactive solids [138] and showed the ability to oxidate minerals [139]. These processes are commercially used in bacterial leaching operations, such as the pretreatment of gold ores which contain arsenopyrite (FeAsS) [139].

Microbial metal reduction can be a strategy for* in situ* and* ex situ* remediation of metal contaminations and wastes. In order to find out the relevance of nanoparticle synthesis and metal reduction, biorecovery of heavy metals, and bioremediation of toxic ones, researchers have investigated the mechanisms of nanoparticle synthesis and bioreduction and focused their attention on reducing agents in bacteria (e.g., proteins and enzymes) and biochemical pathways leading to metal ion reduction. Because of the critical role of these agents, there were more investigations in understanding the role and application of natural and genetically engineered bacterial strains and other microorganisms in bioremediation of toxic metals and radionuclide-contaminated terrestrial environments. Moreover, these microorganisms were capable of mobilization and immobilization of metals [140] and in some cases, the bacteria which could reduce metal ions showed the ability to precipitate metals at nanometer scale. These studies would then lead to check the possibility of genetically engineered microorganisms to overexpress specific reducing molecules and to develop microbial nanoparticle synthesis procedures, which might potentially control size, shape, stability, and yield of NPs. Actually, genetically engineered microorganisms have started being developed in order to increase protein secretion and thus elucidate the most probable reducing agent. For instance, Kang et al. [141] explored for the first time the systematic approach toward the tunable synthesis of semiconductor CdS nanocrystals by genetically engineered* E. coli*. To explore the feasibility of using* E. coli *as a biofactory for the controlled synthesis of CdS nanocrystals, a strain was endowed with the ability to produce phytochelatins (PCs) by expressing the PC synthetase of* S. pombe *(SpPCS). PCs serve as a binding template/nucleation site for the metal ions and stabilize the nanocrystal core against continued aggregation. A feedback-desensitized gamma-glutamylcysteine synthase (GSHI^*^), which catalyzes the synthesis of the PC precursor glutathione (GSH), was cotransformed to enhance the level of PC synthesis 10-fold. After promoted PC synthesis, CdS nanocrystals were produced with a size distribution of 2–6 nm. Moreover, it was proven that glutathione synthetase overexpression in ABLEC* E. coli* strains, in conjunction with metal stress, simultaneously enhanced the biosynthesis of intracellular glutathione and CdS NPs [142]. In another study, stable NPs were recently achieved using the engineering of the* Desulfovibrio desulfuricans* flagellar FliC protein. The introduction of additional cysteine-derived thiol residues in the* E. coli* FliC protein increased Au (III) sorption and reduction on the surface of the flagellar filament and resulted in the synthesis of stabilized gold NPs (20–50 nm). Exhibited biosorption values were about 3 times higher than in the wild type. Wild type flagellar filaments showed fewer gold NPs (~15–50 nm) [34]. Moreover, a strain of* Bacillus licheniformis* was optimized for *α*-amylase production for the synthesis of gold NPs (10–50 nm) [143]. Recombinant strains have been explored for developing more efficient organisms in the* in vivo* synthesis of NPs. For instance, recombinant* E. coli* strains expressing* Arabidopsis thaliana* phytochelatin synthase (AtPCS) and/or* Pseudomonas putida* metal-lothionein (PpMT) were used for the synthesis of Cd, Se, Zn, Te, Cs, Sr, Fe, Co, Ni, Mn, Au, Ag Pr, and Gd NPs. Adjusting the concentrations of supplied metal ions resulted in controlling the size of the metal NPs.

It was reported that the engineered* E. coli* system can be applicable to the biological synthesis of metal NPs [144]. Mutant strains of some bacteria used for synthesis of NPs could help to elucidate the molecules involved in the bioreduction process. For instance, three* E. coli* mutants lacking one or more of the [NiFe] hydrogenases appeared to exhibit altered patterns of Pd (0) deposition as compared to the parental strain. Mutant strains produced highly catalytic Pd NPs by bioreduction of Pd (II). These studies of hydrogenase-deficient mutants suggest that the location of the Pd(0) deposits is in accordance with the subcellular localization of the remaining active hydrogenase and that all three hydrogenases could contribute to Pd (II) reduction* in vivo* [145]. Besides, wild type and three hydrogenase-deficient strains of* Desulfovibrio fructosivorans* were used for the reduction of Pd (II) to Pd(0). The localization of palladium was coincident with the localization of the hydrogenase, suggesting that this enzyme serves as a nucleation site and assists initial Pd nanoparticle growth, probably by supplying the electrons for Pd (II) reduction [146].

In case of* Acidithiobacillus thiooxidans*, gold (I)-thiosulfate was entered into the cell of* A. thiooxidans* as part of a metabolic process [147]. This gold complex was initially decomplexed to Au(I) and thiosulfate (S_2_O_3_^2−^) ions. Thiosulfate was used as energy source and Au(I) was presumably reduced to elemental gold within the bacterial cells. During the late stationary growth phase, the gold NPs which were initially precipitated inside the cells were released from the cells, resulting in the formation of gold particles at the cell surface. Finally, the gold particles in the bulk solution were grown into micrometer-scale wire and octahedral gold [147].

According to Lengke and Southam [148, 149], the precipitation of gold (I)-thiosulfate complex by sulfate-reducing bacteria was caused by three possible mechanisms: (a) iron sulfide formation, (b) localized reducing conditions, and (c) a metabolic process. In the iron system, the formation of iron sulfide generated by sulfate-reducing bacteria could have adsorbed a gold (I)-thiosulfate complex onto freshly forming surfaces, leading to the precipitation of elemental gold. The localized reducing conditions generated by sulfate-reducing bacteria were associated with metabolism. Thiosulfate ion from a gold (I)-thiosulfate complex was initially reduced to hydrogen sulfide (HS^−^), as the end product of metabolism. The release of hydrogen sulfide through the outer membrane pores decreased redox conditions around the cells and caused the precipitation of elemental gold. The precipitation of elemental gold by sulfate-reducing bacteria through metabolic process was initiated when a gold (I)-thiosulfate entered the bacterial cells and was decomplexed to Au(I) and thiosulfate ions. Thiosulfate was used as energy source and Au(I) was presumably reduced to elemental gold within the bacterial cells. During the late stationary growth phase or death phase, the gold NPs which were initially precipitated inside the cells were released into the bulk solution and formed suboctahedral to octahedral, subspherical to spherical aggregates resembling framboids, and ultimately millimeter-thick gold foil at longer experimental duration [148, 149].

Several studies widely reported the act of cytoplasmic and periplasmic hydrogenases produced by microorganisms in metal reduction [38, 72]. In order to show the critical role of these enzymes, researchers used Cu (II) as a selective inhibitor of periplasmic hydrogenases. For example, In case of* D*.* desulfuricans* and* E*.* coli*, partial inhibition of periplasmic hydrogenases with Cu (II) showed that these metal reductase enzymes play a role in Au (III) reduction [34]. Lioyd et al. [115] concluded that periplasmic hydrogenases were possibly responsible for Pd (II) reduction and inhibited by Cu (II). Furthermore, Au (III) reduction was done in the presence of H_2_ (as the electron donor) using microorganisms such as* T*.* maritima*,* S*.* alga*,* D*.* vulgaris*,* G*.* ferrireducens*,* D*.* desulfuricans*, and* E*.* coli*. Possibly, hydrogenases play an important role in Au (III) reduction [28, 34, 84], but more investigations were needed to know exact mechanisms of these reductions. Moreover, it was reported that hydrogenase is involved in U^+6^ reduction by* Micrococcus lactyliticus* [150], in addition to Se^+6^ reduction by* Clostridium pasteurianum* [151]. Hydrogenases from the sulfate-reducing bacteria have been shown to be capable of reducing Tc^+7^ and Cr^+6^ [152]. In another study, it was reported that the hydrogenases isolated from phototrophic bacteria were able to reduce Ni^+2^ to Ni^0^ under an H_2_ atmosphere [153]. In case of* S. algae*, the microbial reduction of gold ions was dependent on the presence of a specific electron donor, the molecular H_2_. It was concluded that the* S. algae* hydrogenase catalyzes the activation of molecular H_2_ using the molecule as the electron donor according to the following reaction:

(1) $$\begin{array}{c} {\text{H}}_{2}⟶2{\text{H}}^{+}+2{\text{e}}^{-}. \end{array}$$

Therefore, the* S. algae* cells are likely to transfer electrons to AuCl_4_^−^ ions, reducing them to gold metals. Consider

(2) $$\begin{array}{c} {{\text{AuCl}}_{4}}^{-}+3{\text{e}}^{-}⟶\text{Au}\;+4{\text{Cl}}^{-}. \end{array}$$

In some cases, researchers purified proteins which were believed to be responsible for nanoparticle synthesis. Matsunaga et al. [154] have shown that MagA gene and its protein (isolated from* Magnetospirillum* sp. AMB-1) were required for biomagnetic nanoparticle formation. Magnetotactic bacteria (e.g.* M*.* magnetotacticum* and* M*.* gryphiswaldense*), contain magnetosome membrane (MM) proteins which play an important role in magnetite biomineralization. Thus, researchers have focused on identification of these proteins and their genes [155, 156]. Recent molecular studies including genome sequence, mutagenesis, gene expression, and proteome analyses indicated a number of genes and proteins which play critical roles for bacterial magnetic particles biomineralization [157]. Moisescu et al. [158] have studied the chemical composition and microstructural characteristics of bacterial magnetosomes extracted from the magnetotactic bacterial strain* M. gryphiswaldense*. They reported the produced cuboctahedral magnetite particles with an average diameter of 46 ± 6.8 nm. The particles exhibited a high chemical purity (exclusively Fe_3_O_4_) and the majorities fall within the single-magnetic-domain range.

In cases of* Rhodopseudomonas capsulata* and* Stenotrophomonas maltophilia*, the authors believed that the specific NADPH-dependent enzyme present in the isolated strains reduced Au^+3^ to Au^0^ through an electron shuttling mechanism, leading to the synthesis of monodispersed NPs. A two-step process is needed to reduce gold ions. During the first step, the AuCl_4_^−^ ions are reduced to the Au^+^ species. Then, the latter product is reduced by NADHP to a metallic gold [77, 78].

Mechanisms of gold accumulation by the cyanobacteria studied from gold chloride initially promoted the precipitation of amorphous gold sulfide at the cell walls and finally deposited metallic gold in octahedral form near the cell surfaces and in solutions [43]. Moreover, it was reported that the formation of gold NPs by* Plectonema boryanum* UTEX 485 occurred by three possible mechanisms involving iron sulfide, localized reducing conditions, and metabolism [148]. Lengke et al. [118] biosynthesized spherical platinum NPs using cyanobacterium,* P. boryanum* UTEX 485. The addition of PtCl4^0^ to the bacterial culture initially promoted the precipitation of Pt^+2^-organic metal as spherical NPs in the solution, which then dispersed within the bacterial cells. The cyanobacteria were immediately killed either by the PtCl4^0^, by the acidic pH, or by the elevated temperatures (~60–180°C); the resulting release of organics caused further precipitation of platinum. With an increase in temperature, the Pt^2^-organic NPs were recrystallized and formed NPs consisting of a platinum metal. Riddin et al. [159] reported the bioreduction of Pt^+4^ into the Pt^0^ NPs. A mixed and uncharacterized consortium of sulphate-reducing bacteria was used to investigate the mechanism in the platinum nanoparticle formation. It was shown that two different hydrogenase enzymes were involved. First, the Pt^+4^ ions were reduced to Pt^+2^ by an oxygen-sensitive cytoplasmic hydrogenase. Then, the formed ions were reduced to Pt^0^ NPs by a periplasmic hydrogenase that was oxygen tolerant and was inhibited by Cu^+2^.

To control the morphologies and sizes of NPs, there were several investigations focused on using proteins. Interestingly, association of proteins with spheroidal aggregates of biogenic zinc sulfide nanocrystals documented that extracellular proteins originated from microorganisms could limit the biogenic NPs [160]. Controlled formation of magnetite crystals with uniformed size was achieved in the presence of Mms6 (a small acidic protein isolated from* Magnetospirillum magneticum *AMB-1) [161]. The average size of magnetite crystals synthesized in the presence of Mms6 was about 20.2 + 4.0 nm. But in the absence of Mms6, the synthesized magnetite crystals were about 32.4 + 9.1 nm. Therefore, the crystals synthesized with Mms6 were smaller than crystals produced without Mms6 and were distributed over a narrower range than crystals synthesized in the absence of the protein. Mms6 also promoted formation of uniform, isomorphic, superparamagnetic nanocrystals [162]. With a bioinspired method, Prozorov et al. [163] have reported the use of the recombinant Mms6 protein for synthesis of uniform, well-defined CoFe_2_O_4_ nanocrystals in vitro. In order to template hierarchical CoFe_2_O_4_ nanostructures, a recombinant polyhistidine-tagged full-length Mms6 protein and a synthetic C-terminal domain of this protein were covalently attached to triblock copolymers (poloxamers).

In case of* Klebsiella pneumoniae*, it was reported that no formation of silver NPs by the supernatant was observed when the procedure took place in the dark. The visible-light emission can significantly cause the synthesis of NPs. It seems that in this case, the silver ions reduction was mainly due to conjugation shuttles with the participation of the reductase. Thus, it appears that the cell-associated nitroreductase enzymes may be involved in the photoreduction of silver ions [164]. In addition, mechanisms of cadmium sulfide nanocrystals synthesis by* E*.* coli* cells were explained through the control experiments (incubation of CdCl_2_ and Na_2_S without bacterial cells) which indicated that nanocrystals were not synthesized outside the cells and then transported into the cells [30]. These experiments have shown that CdS nanocrystals could be synthesized following Cd^2+^ and S^2−^ ions transported into the cells. In case of zinc sulfide (ZnS), the NPs could be formed intracellularly through the biological synthetic method suggested by Bai et al. [48]. They explained that soluble sulfate diffused into immobilized beads and then was carried to the interior membrane of* R*.* sphaeroides* cell facilitated by sulfate permease. After that, ATP sulfurylase and phosphoadenosine phosphosulfate reductase reduced sulfate to sulfite, and sulfite reductase reduced sulfite to sulfide which reacted with O-acetylserine to synthesize cysteine via O-acetylserine thiol lyase. Then, cysteine produced S^2−^ by a cysteine desulfhydrase in presence of zinc. After this process, spherical ZnS NPs were synthesized following the reaction of S^2−^ with the soluble zinc salt. These NPs were discharged from immobilized* R*.* sphaeroides* cells to the solution.

## 4. Future Prospects 

Major drawbacks associated with the biosynthesis of NPs using bacteria are tedious purification steps and poor understanding of the mechanisms. The important challenges frequently encountered in the biosynthesis of NPs are to control the shape and size of the particles and to achieve the monodispersity in solution phase. It seems that several important technical challenges must be overcome before this green bio-based method will be a successful and competitive alternative for industrial synthesis of NPs. An important challenge is scaling up for production level processing. Furthermore, little is known about the mechanistic aspects, and information in this regard is necessary for economic and rational development of nanoparticle biosynthesis. The important aspects which might be considered in the process of producing well-characterized NPs are as follows.

1. *Selection of the best bacteria*. In order to choose the best candidates, researchers have focused on some important intrinsic properties of the bacteria including growth rate, enzyme activities, and biochemical pathways. Choosing a good candidate for nanoparticle production depends on the application we expect from the resulting NPs. For instance, one may need to synthesis NPs with smaller sizes or specific shapes, or it might be important to synthesize NPs within less time [6, 11].
2. *Selection of the biocatalyst state*. It seems that the bacterial enzymes (the biocatalysts) are the major agents in nanoparticle synthesis. The biocatalysts can be used as either of whole cells, crude enzymes, and purified enzymes. It seems that using culture supernatant or cell extract of the cell could increase the rate of reaction. However these NPs did not show the long term stability. Moreover, release of NPs from the cells was an important aspect which might be considered in case of intracellularly produced NPs. Most of the reactions responsible for nanoparticle synthesis seem to be bioreductions. In bioreduction processes, we need the coenzymes (e.g., NADH, NADPH, FAD, etc.) to be supplied in stoichiometric amounts. As they are expensive, the use of whole cells is preferred, because the coenzymes will be recycled during the pathways in live whole cells [11].
3. *Optimal conditions for cell growth and enzyme activity*. We need to produce greater amounts of the enzymes which can be accomplished by synthesis of more biomass. Thus, optimization of the growth conditions is very important. The nutrients, inoculum size, pH, light, temperature, buffer strength, and mixing speed should be optimized. Induction of the responsible enzymes seems to be crucial, as well. The presence of the substrates or related compounds in subtoxic levels from the beginning of the growth would increase the activity. Harvesting time is important in case of using whole cells and crude enzymes. Therefore, it might be necessary to monitor the enzyme activity during the time course of growth [11].
4. *Optimal reaction conditions*. It is better to harvest the cells (the biocatalysts) to remove unwanted residual nutrients and metabolites in order to avoid adverse reactions and provide cleaner medium for better and easier analysis. In order to use bacteria for synthesis of nanoparticle in industrial scale, the yield and the production rate are important issues to be considered. Therefore, we need to optimize the bioreduction conditions in the reaction mixture. The substrate concentration (to be in subtoxic level for the biocatalyst), the biocatalyst concentration, the electron donor (and its concentration), exposure time, pH, temperature, buffer strength, mixing speed, and light need to be optimized. The researchers have used some complementary factors such as visible light or microwave irradiation and boiling which could affect the morphology, size, and rate of reaction. It seems that by optimization of these critical parameters, highly stable NPs with desired sizes and morphologies can be achieved. In addition, purification, isolation, and stabilization of the produced NPs are very important, and challenges in this regard must be overcome. Researchers have focused their attention on finding optimal reaction conditions and cellular mechanisms involved in the bioreduction of metal ions and synthesis of NPs [6–12, 165].
5. *Extraction and purification processes*. The extraction and purification of the produced metal NPs from bacteria (intercellular or extracellular synthesis) for further applications are not well investigated, but studies are moving toward solving these problems and finding the best ways. In order to release the intracellularly produced NPs, additional processing steps such as ultrasound treatment or reaction with suitable detergents are required. This can be exploited in the recovery of precious metals from mine wastes and metal leachates. Biomatrixed metal NPs could also be used as catalysts in various chemical reactions. This will help to retain the NPs for continuous usage in bioreactors. Physicochemical methods including freeze-thawing, heating processes, and osmotic shock can be used to extract the produced NPs from the cells. But, it seems that these methods may interfere with the structure of NPs, and aggregation, precipitation, and sedimentation could happen. These may change the shape and size of NPs and interfere with the suitable properties of them. Moreover, enzymatic lysis of the microbial cells containing intracellular NPs can be used, but this method is expensive and it cannot be used in up-scalable and industrial production of NPs. It seems that surfactants and organic solvents can be used for both extraction and stabilization of NPs, but these chemical materials are toxic, expensive, and hazardous. It should be noted that in case of extracellular production of nanoparticle, centrifuge could be used for extraction and purification of NPs, but aggregation might happen.
6. *Stabilization of the produced NPs.* Researchers have illustrated that the NPs produced by these ecofriendly bio-based approaches, showed an interesting stability without any aggregations even for many weeks in room temperature [19, 166]. The stability of these NPs might be due to the proteins and enzymes secreted by the microorganisms. Thus, it seems that these green approaches can be used for synthesis of highly stable NPs.
7. *Scaling up the laboratory process to the industrial scale*. Optimization of the reaction conditions may lead to the enhanced biosynthesis of NPs. Biological protocols could be used for synthesis of highly stable and well-characterized NPs when critical aspects, such as types of organisms, inheritable and genetical properties of organisms, optimal conditions for cell growth and enzyme activity, optimal reaction conditions, and selection of the biocatalyst state have been considered. Size and morphology of the NPs can be controlled by altering the aforementioned reaction conditions (optimal reaction conditions section). Industrial scale synthesis of metal NPs using biomass needs some processes, including seed culture, inoculation of the seed into the biomass, harvesting the cells, synthesis of NPs by adding metal ions to the cells, separation of cells by filtration, homogenization of the cells to isolate the produced NPs, stabilization of the NPs, product formulation, and quality control [6–12, 165].

## 5. Conclusion

Bio-based approaches are still in the development stages, and stability and aggregation of the biosynthesized NPs, control of crystal growth, shape, size, and size distribution are the most important experienced problems. Furthermore, biologically synthesized NPs in comparison with chemically synthesized ones are more polydisperse. The properties of NPs can be controlled by optimization of important parameters which control the growth condition of organisms, cellular activities, and enzymatic processes (optimization of growth and reaction conditions).

Mechanistic aspects have not been clearly and deeply described and discussed. Thus, more elaborated studies are needed to know the exact mechanisms of reaction and identify the enzymes and proteins which involve nanoparticle biosynthesis. The large-scale synthesis of NPs using bacteria is interesting because it does not need any hazardous, toxic, and expensive chemical materials for synthesis and stabilization processes. It seems that by optimizing the reaction conditions and selecting the best bacteria, these natural nanofactories can be used in the synthesis of stable NPs with well-defined sizes, morphologies, and compositions.

## Figures and Tables

**Table 1:** Green biosynthesis of NPs using bacteria.

Bacteria	Nanoparticle	Size (nm)	Morphology	References
*Aeromonas sp*. SH10	Silver	6.4	—	[17]
*Bacillus cereus *	Silver	20–40	Spherical	[18]
*Bacillus megatherium* D01	Gold	1.9 ± 0.8	Spherical	[19]
*Bacillus subtilis *168	Gold	5–25	Octahedral	[20, 21]
*Bacillus subtilis *	Silver	5–50	Spherical and triangular	[22]
*Clostridium thermoaceticum *	Cadmium sulfide	—	Amorphous	[23]
*Corynebacterium *sp. SH09	Silver	10–15	—	[24]
*Desulfobacteraceae *	Zinc sulfide	2–5	Spherical	[25]
*Desulfovibrio desulfuricans *	Palladium and selenium	—	—	[26, 27]
*Desulfovibrio vulgaris *	Gold, uranium, and chromium	—	—	[28]
*Desulfovibrio magneticus* strain RS-1	Magnetite	Up to 30	Crystalline	[29]
*Enterobacter cloacae *	Silver and selenium	—	—	[26]
*Escherichia coli *	Cadmium sulfide	2–5	Wurtzite crystal	[30]
*Escherichia coli *	Silver	8-9	Spherical	[31]
*Escherichia coli* DH5*α*	Silver	10–100	Spherical	[32]
*Escherichia coli *DH5*α*	Gold	25 ± 8	Spherical, triangular, and quasi-hexagonal	[33]
*Escherichia coli *MC4100	Gold	Less than 10 to 50	Spherical, triangular, hexagonal, and rod shape	[34]
*Geobacillus *sp.	Gold	5–50	Quasi-hexagonal	[35]
*Geovibrio ferrireducens *	Gold	—	—	[28]
*Klebsiella aerogenes *	Cadmium sulfide	20–200	Crystalline	[36]
*Klebsiella pneumonia *	Silver	28.2–122 (average size of 52.2)	Spherical	[37]
*Lactobacillus* strains	Gold	20–50 and above 100	Crystalline, hexagonal, triangular, and cluster	[38]
*Lactobacillus* strains	Silver	15–500	Crystalline, hexagonal, triangular, and cluster	[38]
*Lactobacillus* strains	Silver-gold alloys	100–300	Crystalline and cluster	[38]
*Lactobacillus* strains	Titanium	40–60	Spherical	[39]
*Lactobacillus casei* subsp. *casei *	Silver	25–50	Spherical	[12]
*Magnetospirillum magnetotacticum *	Magnetite	—	Cluster (folded-chain and flux-closure ring)	[40]
*Nocardiopsis* sp. MBRC-1	Silver	~45	Spherical	[41]
*Plectonema boryanum *UTEX 485	Gold	10–25 and ~1–10 and 10 to 6000	Cubic and octahedral Platelet	[42, 43]
*Pseudomonas aeruginosa *	Gold	15–30	—	[44]
*Pseudomonas aeruginosa *	Lanthanum	—	Crystalline and needle-like	[12, 14]
*Pseudomonas fluorescens *	Gold	50–70	Spherical	[45]
*Pseudomonas putida* NCIM 2650	Silver	~70	Spherical	[46]
*Pseudomonas* *stutzeri* AG259	Silver	35–46 and up to 200	Hexagonal, equilateral triangle, crystalline silver, and monoclinic silver sulfide acanthite	[47]
*Rhodobacter sphaeroides *	Zinc sulfide	Average diameter of 8	Spherical	[48]
*Rhodopseudomonas capsulata *	Gold	10–20	Nanoplate and spherical	[15, 49]
*Rhodopseudomonas* *palustris *	Cadmium sulfide	8.01 ± 0.25	Crystalline, face-centered cubic	[50]
*Serratia nematodiphila *	Silver	10–31	spherical, and crystalline	[51]
*Shewanella algae *	Platinum	5	elemental	[52]
*Shewanella algae* strain BRY	Gold	Various sizes changed with pH	—	[28]
*Shewanella putrefaciens* (Gs-15)	Magnetite	10–50	Fine-grained crystal	[53]
*Thermoanaerobacter ethanolicus* TOR-39	Magnetite, cobalt, nickel, and chromium	—	Octahedral	[13, 54]

## References

[1] Bhattacharya D., Gupta R. K. (2005). Nanotechnology and potential of microorganisms. *Critical Reviews in Biotechnology*.

[2] Goodsell D. (2004). *Bionanotechnology: Lessons from Nature*.

[3] Paull R., Wolfe J., Hébert P., Sinkula M. (2003). Investing in nanotechnology. *Nature Biotechnology*.

[4] Salata O. V. (2004). Applications of nanoparticles in biology and medicine. *Journal of Nanobiotechnology*.

[5] Sastry M., Ahmad A., Islam Khan M., Kumar R. (2003). Biosynthesis of metal nanoparticles using fungi and actinomycete. *Current Science*.

[6] Iravani S. (2011). Green synthesis of metal nanoparticles using plants. *Green Chemistry*.

[7] Iravani S., Korbekandi H., Mirmohammadi S. V., Mekanik H. (2014). Plants in nanoparticle synthesis. *Reviews in Advanced Sciences and Engineering*.

[8] Iravani S., Zolfaghari B. (2013). Green synthesis of silver nanoparticles using *Pinus eldarica* bark extract. *Biomed Research International*.

[9] Korbekandi H., Ashari Z., Iravani S., Abbasi S. (2013). Optimization of biological synthesis of silver nanoparticles using *Fusarium oxysporum*. *Iranian Journal of Pharmaceutical Research*.

[10] Korbekandi H., Iravani S., Rai M., Posten C. (2013). Biological synthesis of nanoparticles using algae. *Green Biosynthesis of Nanoparticles: Mechanisms and Applications*.

[11] Korbekandi H., Iravani S., Abbasi S. (2009). Production of nanoparticles using organisms. *Critical Reviews in Biotechnology*.

[12] Korbekandi H., Iravani S., Abbasi S. (2012). Optimization of biological synthesis of silver nanoparticles using *Lactobacillus casei subsp. casei*. *Journal of Chemical Technology & Biotechnology*.

[13] Klaus-Joerger T., Joerger R., Olsson E., Granqvist C. (2001). Bacteria as workers in the living factory: metal-accumulating bacteria and their potential for materials science. *Trends in Biotechnology*.

[14] Mullen M. D., Wolf D. C., Ferris F. G., Beveridge T. J., Flemming C. A., Bailey G. W. (1989). Bacterial sorption of heavy metals. *Applied and Environmental Microbiology*.

[15] He S., Guo Z., Zhang Y., Zhang S., Wang J., Gu N. (2007). Biosynthesis of gold nanoparticles using the bacteria *Rhodopseudomonas capsulata*. *Materials Letters*.

[16] Lengke M. F., Fleet M. E., Southam G. (2007). Biosynthesis of silver nanoparticles by filamentous cyanobacteria from a silver(I) nitrate complex. *Langmuir*.

[17] Rai A., Singh A., Ahmad A., Sastry M. (2006). Role of halide ions and temperature on the morphology of biologically synthesized gold nanotriangles. *Langmuir*.

[18] Sunkar S., Nachiyar C. V. (2012). Biogenesis of antibacterial silver nanoparticles using the endophytic bacterium *Bacillus cereus* isolated from *Garcinia xanthochymus*. *Asian Pacific Journal of Tropical Biomedicine*.

[19] Wen L., Lin Z., Gu P. (2009). Extracellular biosynthesis of monodispersed gold nanoparticles by a SAM capping route. *Journal of Nanoparticle Research*.

[20] Beveridge T. J., Murray R. G. E. (1980). Sites of metal deposition in the cell wall of *Bacillus subtilis*. *Journal of Bacteriology*.

[21] Southam G., Beveridge T. J. (1994). The in vitro formation of placer gold by bacteria. *Geochimica et Cosmochimica Acta*.

[22] Saifuddin N., Wong C. W., Yasumira A. A. N. (2009). Rapid biosynthesis of silver nanoparticles using culture supernatant of bacteria with microwave irradiation. *E-Journal of Chemistry*.

[23] Cunningham D. P., Lundie L. L. (1993). Precipitation of cadmium by *Clostridium thermoaceticum*. *Applied and Environmental Microbiology*.

[24] Zhang H. R., Li Q. B., Lu Y. H. (2005). Biosorption and bioreduction of diamine silver complex by *Corynebacterium*. *Journal of Chemical Technology and Biotechnology*.

[25] Labrenz M., Druschel G. K., Thomsen-Ebert T. (2000). Formation of sphalerite (ZnS) deposits in natural biofilms of sulfate-reducing bacteria. *Science*.

[26] Kessi J., Ramuz M., Wehrli E., Spycher M., Bachofen R. (1999). Reduction of selenite and detoxification of elemental selenium by the phototrophic bacterium *Rhodospirillum rubrum*. *Applied and Environmental Microbiology*.

[27] Yong P., Rowson N. A., Farr J. P. G., Harris I. R., Macaskie L. E. (2002). Bioreduction and biocrystallization of palladium by *Desulfovibrio desulfuricans* NCIMB 8307. *Biotechnology and Bioengineering*.

[28] Kashefi K., Tor J. M., Nevin K. P., Lovley D. R. (2001). Reductive precipitation of gold by dissimilatory Fe(III)-reducing bacteria and archaea. *Applied and Environmental Microbiology*.

[29] Pósfai M., Moskowitz B. M., Arató B. (2006). Properties of intracellular magnetite crystals produced by *Desulfovibrio magneticus* strain RS-1. *Earth and Planetary Science Letters*.

[30] Sweeney R. Y., Mao C., Gao X. (2004). Bacterial biosynthesis of cadmium sulfide nanocrystals. *Chemistry and Biology*.

[31] Mahanty A., Bosu R., Panda P., Netam S. P., Sarkar B. (2013). Microwave assisted rapid combinatorial synthesis of silver nanoparticles using *E. coli* culture supernatant. *International Journal of Pharma and Bio Sciences*.

[32] Ghorbani H. R. (2013). Biosynthesis of silver nanoparticles by *Escherichia coli*. *Asian Journal of Chemistry*.

[33] Du L., Jiang H., Liu X., Wang E. (2007). Biosynthesis of gold nanoparticles assisted by Escherichia coli DH5*α* and its application on direct electrochemistry of hemoglobin. *Electrochemistry Communications*.

[34] Deplanche K., Macaskie L. E. (2008). Biorecovery of gold by *Escherichia coli* and *Desulfovibrio desulfuricans*. *Biotechnology and Bioengineering*.

[35] Correa-Llantén D. N., Muñoz-Ibacache S. A., Castro M. E., Muñoz P. A., Blamey J. M. (2013). Gold nanoparticles synthesized by *Geobacillus* sp. strain ID17 a thermophilic bacterium isolated from Deception Island, Antarctica. *Microbial Cell Factories*.

[36] Holmes J. D., Smith P. R., Evans-Gowing R., Richardson D. J., Russell D. A., Sodeau J. R. (1995). Energy-dispersive X-ray analysis of the extracellular cadmium sulfide crystallites of *Klebsiella aerogenes*. *Archives of Microbiology*.

[37] Shahverdi A. R., Minaeian S., Shahverdi H. R., Jamalifar H., Nohi A. (2007). Rapid synthesis of silver nanoparticles using culture supernatants of Enterobacteria: a novel biological approach. *Process Biochemistry*.

[38] Nair B., Pradeep T. (2002). Coalescence of nanoclusters and formation of submicron crystallites assisted by *Lactobacillus* strains. *Crystal Growth and Design*.

[39] Prasad K., Jha A. K., Kulkarni A. R. (2007). Lactobacillus assisted synthesis of titanium nanoparticles. *Nanoscale Research Letters*.

[40] Philipse A. P., Maas D. (2002). Magnetic colloids from magnetotactic bacteria: chain formation and colloidal stability. *Langmuir*.

[41] Manivasagan P., Venkatesan J., Senthilkumar K., Sivakumar K., Kim S. (2013). Biosynthesis, antimicrobial and cytotoxic effect of silver nanoparticles using a novel *Nocardiopsis* sp. MBRC-1. *BioMed Research International*.

[42] Lengke M. F., Fleet M. E., Southam G. (2006). Morphology of gold nanoparticles synthesized by filamentous cyanobacteria from gold(I)-Thiosulfate and gold(III)-chloride complexes. *Langmuir*.

[43] Lengke M. F., Ravel B., Fleet M. E., Wanger G., Gordon R. A., Southam G. (2006). Mechanisms of gold bioaccumulation by filamentous cyanobacteria from gold(III)-chloride complex. *Environmental Science and Technology*.

[44] Husseiny M. I., El-Aziz M. A., Badr Y., Mahmoud M. A. (2007). Biosynthesis of gold nanoparticles using *Pseudomonas aeruginosa*. *Spectrochimica Acta A: Molecular and Biomolecular Spectroscopy*.

[45] Rajasree S. R., Suman T. Y. (2012). Extracellular biosynthesis of gold nanoparticles using a gram negative bacterium *Pseudomonas fluorescens*. *Asian Pacific Journal of Tropical Disease*.

[46] Thamilselvi V., Radha K. V. (2013). Synthesis of silver nanoparticles from *Pseudomonas putida* NCIM 2650 in silver nitrate supplemented growth medium and optimization using response surface methodology. *Digest Journal of Nanomaterials and Biostructures*.

[47] Haefeli C., Franklin C., Hardy K. (1984). Plasmid-determined silver resistance in Pseudomonas stutzeri isolated from a silver mine. *Journal of Bacteriology*.

[48] Bai H., Zhang Z., Gong J. (2006). Biological synthesis of semiconductor zinc sulfide nanoparticles by immobilized Rhodobacter sphaeroides. *Biotechnology Letters*.

[49] He S., Zhang Y., Guo Z., Gu N. (2008). Biological synthesis of gold nanowires using extract of *Rhodopseudomonas capsulata*. *Biotechnology Progress*.

[50] Bai H. J., Zhang Z. M., Guo Y., Yang G. E. (2009). Biosynthesis of cadmium sulfide nanoparticles by photosynthetic bacteria *Rhodopseudomonas palustris*. *Colloids and Surfaces B: Biointerfaces*.

[51] Malarkodi C., Rajeshkumar S., Paulkumar K., Vanaja M., Jobitha G. D. G., Annadurai G. (2013). Bactericidal activity of bio mediated silver nanoparticles synthesized by *Serratia nematodiphila*. *Drug Invention Today*.

[52] Konishi Y., Ohno K., Saitoh N. (2007). Bioreductive deposition of platinum nanoparticles on the bacterium *Shewanella algae*. *Journal of Biotechnology*.

[53] Lovley D. R., Stolz J. F., Nord G. L., Phillips E. J. P. (1987). Anaerobic production of magnetite by a dissimilatory iron-reducing microorganism. *Nature*.

[54] Yeary L. W., Moon J., Love L. J., Thompson J. R., Rawn C. J., Phelps T. J. (2005). Magnetic properties of biosynthesized magnetite nanoparticles. *IEEE Transactions on Magnetics*.

[55] Bridges K., Kidson A., Lowbury E. J. L., Wilkins M. D. (1979). Gentamicin- and silver-resistant Pseudomonas in a burns unit. *British Medical Journal*.

[56] Brock T. D., Gustafson J. (1976). Ferric iron reduction by sulfur- and iron-oxidizing bacteria.. *Applied and Environmental Microbiology*.

[57] Taylor D. E. (1999). Bacterial tellurite resistance. *Trends in Microbiology*.

[58] Lloyd J. R., Ridley J., Khizniak T., Lyalikova N. N., Macaskie L. E. (1999). Reduction of technetium by *Desulfovibrio desulfuricans*: biocatalyst characterization and use in a flowthrough bioreactor. *Applied and Environmental Microbiology*.

[59] Kalishwaralal K., Deepak V., Ramkumarpandian S., Nellaiah H., Sangiliyandi G. (2008). Extracellular biosynthesis of silver nanoparticles by the culture supernatant of *Bacillus licheniformis*. *Materials Letters*.

[60] Kalimuthu K., Suresh Babu R., Venkataraman D., Bilal M., Gurunathan S. (2008). Biosynthesis of silver nanocrystals by *Bacillus licheniformis*. *Colloids and Surfaces B: Biointerfaces*.

[61] Priyadarshini S., Gopinath V., Meera Priyadharsshini N., MubarakAli D., Velusamy P. (2013). Synthesis of anisotropic silver nanoparticles using novel strain, *Bacillus flexus* and its biomedical application. *Colloids and Surfaces B: Biointerfaces*.

[62] Wei X., Luo M., Li W. (2012). Synthesis of silver nanoparticles by solar irradiation of cell-free *Bacillus amyloliquefaciens* extracts and AgNO_3_. *Bioresource Technology*.

[63] Slawson R. M., Van Dyke M. I., Lee H., Trevors J. T. (1992). Germanium and silver resistance, accumulation, and toxicity in microorganisms. *Plasmid*.

[64] Klaus T., Joerger R., Olsson E., Granqvist C. (1999). Silver-based crystalline nanoparticles, microbially fabricated. *Proceedings of the National Academy of Sciences of the United States of America*.

[65] Shivaji S., Madhu S., Singh S. (2011). Extracellular synthesis of antibacterial silver nanoparticles using psychrophilic bacteria. *Process Biochemistry*.

[66] Lovley D. R. (1991). Dissimilatory Fe(III) and Mn(IV) reduction. *Microbiological Reviews*.

[67] Suresh A. K., Pelletier D. A., Wang W. (2010). Silver nanocrystallites: biofabrication using shewanella oneidensis, and an evaluation of their comparative toxicity on gram-negative and gram-positive bacteria. *Environmental Science and Technology*.

[68] Mokhtari N., Daneshpajouh S., Seyedbagheri S. (2009). Biological synthesis of very small silver nanoparticles by culture supernatant of Klebsiella pneumonia: the effects of visible-light irradiation and the liquid mixing process. *Materials Research Bulletin*.

[69] Fu M., Li Q., Sun D. (2006). Rapid preparation process of silver nanoparticles by bioreduction and their characterizations. *Chinese Journal of Chemical Engineering*.

[70] Kapoor S. (1998). Preparation, characterization, and surface modification of silver particles. *Langmuir*.

[71] Satyanarayana R. A., Chen C., Jean J. (2010). Biological synthesis of gold and silver nanoparticles mediated by the bacteria *Bacillus subtilis*. *Journal of Nanoscience and Nanotechnology*.

[72] Bowman J. P., McCammon S. A., Nichols D. S. (1997). *Shewanella gelidimarina* sp. nov. and *Shewanella frigidimarina* sp. nov., novel antarctic species with the ability to produce eicosapentaenoic acid (20:5*ω*3) and grow anaerobically by dissimilatory fe(III) reduction. *International Journal of Systematic Bacteriology*.

[73] Bozal N., Montes M. J., Tudela E., Jiménez F., Guinea J. (2002). *Shewanella frigidimarina* and *Shewanella livingstonensis* sp. nov. isolated from Antarctic coastal areas. *International Journal of Systematic and Evolutionary Microbiology*.

[74] Ivanova E. P., Sawabe T., Gorshkova N. M. (2001). *Shewanella japonica* sp. nov.. *International Journal of Systematic and Evolutionary Microbiology*.

[75] Ivanova E. P., Nedashkovskaya O. I., Zhukova N. V., Nicolau D. V., Christen R., Mikhailov V. V. (2003). *Shewanella waksmanii* sp. nov., isolated from a sipunculla (*Phascolosoma japonicum*). *International Journal of Systematic and Evolutionary Microbiology*.

[76] Ivanova E. P., Sawabe T., Hayashi K. (2003). *Shewanella fidelis* sp. nov., isolated from sediments and sea water. *International Journal of Systematic and Evolutionary Microbiology*.

[77] Nogi Y., Kato C., Horikoshi K. (1998). Taxonomic studies of deep-sea barophilic Shewanella strains and description of *Shewanella violacea* sp. nov.. *Archives of Microbiology*.

[78] Satomi M., Oikawa H., Yano Y. (2003). *Shewanella marinintestina* sp. nov., *Shewanella schlegeliana* sp. nov. and *Shewanella sairae* sp. nov., novel eicosapentaenic-acid-producing marine bacteria isolated from sea-animal intestines. *International Journal of Systematic and Evolutionary Microbiology*.

[79] Venkateswaran K., Moser D. P., Dollhopf M. E. (1999). Polyphasic taxonomy of the genus Shewanella and description of *Shewanella oneidensis* sp. nov.. *International Journal of Systematic Bacteriology*.

[80] Zhao J. S., Manno D., Beaulieu C., Paquet L., Hawari J. (2005). *Shewanella sediminis* sp. nov., a novel Na^+^- requiring and hexahydro-1,3,5-trinitro-1,3,5-triazine-degrading bacterium from marine sediment. *International Journal of Systematic and Evolutionary Microbiology*.

[81] Kim D., Kang C., Lee C. S. (2006). Treatment failure due to emergence of resistance to carbapenem during therapy for Shewanella algae bacteremia. *Journal of Clinical Microbiology*.

[82] Konishi Y., Tsukiyama T., Tachimi T., Saitoh N., Nomura T., Nagamine S. (2007). Microbial deposition of gold nanoparticles by the metal-reducing bacterium Shewanella algae. *Electrochimica Acta*.

[83] Caccavo F., Blakemore R. P., Lovley D. R. (1992). A hydrogen-oxidizing, Fe(III)-reducing microorganism from the Great Bay estuary, New Hampshire. *Applied and Environmental Microbiology*.

[84] Konishi Y., Tsukiyama T., Ohno K., Saitoh N., Nomura T., Nagamine S. (2006). Intracellular recovery of gold by microbial reduction of AuCl_4_
^−^ ions using the anaerobic bacterium Shewanella algae. *Hydrometallurgy*.

[85] Fernando L. M., Merca F. E., Paterno E. S. (2013). Biogenic synthesis of gold nanoparticles by plant-growth-promoting bacteria isolated from philippine soils. *Philippine Agricultural Scientist*.

[86] Arató B., Schüler D., Flies C., Bazylinski D., Frankel R., Buseck P. (2004). Intracellular magnetite and extracellular hematite produced by Desulfovibrio magneticus strain RS-1. *Geophysical Research Abstracts*.

[87] Roh Y., Lauf R. J., McMillan A. D. (2001). Microbial synthesis and the characterization of metal-substituted magnetites. *Solid State Communications*.

[88] Lee H., Purdon A. M., Chu V., Westervelt R. M. (2004). Controlled assembly of magnetic nanoparticles from magnetotactic bacteria using microelectromagnets arrays. *Nano Letters*.

[89] Baedecker M. J., Back W. (1979). Modern marine sediments as a natural analog to the chemically stressed environment of a landfill. *Journal of Hydrology*.

[90] Lovley D. (1987). Organic matter mineralization with the reduction of ferric iron. *Geomicrobiology Journal*.

[91] Graybeal A. L., Heath G. R. (1984). Remobilization of transition metals in surficial pelagic sediments from the eastern Pacific. *Geochimica et Cosmochimica Acta*.

[92] Bostrom B., Jansson M., Forsberg C. (1982). Phosphorus release from lake sediments. *Archiv für Hydrobiologie—Beiheft Ergebnisse der Limnologie*.

[93] Watson J. H. P., Ellwood D. C., Soper A. K., Charnock J. (1999). Nanosized strongly-magnetic bacterially-produced iron sulfide materials. *Journal of Magnetism and Magnetic Materials*.

[94] Watson J. H. P., Croudace I. W., Warwick P. E., James P. A. B., Charnock J. M., Ellwoods D. C. (2001). Adsorption of radioactive metals by strongly magnetic iron sulfide nanoparticles produced by sulfate-reducing bacteria. *Separation Science and Technology*.

[95] Lovley D. R., Phillips E. J. P. (1988). Novel mode of microbial energy metabolism: organic carbon oxidation coupled to dissimilatory reduction of iron or manganese. *Applied & Environmental Microbiology*.

[96] Lovley D. R., Phillips E. J. P., Lonergan D. J. (1989). Hydrogen and formate oxidation coupled to dissimilatory reduction of iron or manganese by *Alteromonas putrefaciens*. *Applied and Environmental Microbiology*.

[97] Lovley D. R., Phillips E. J. P., Lonergan D. J., Widman P. K. (1995). Fe(III) and S0 reduction by *Pelobacter carbinolicus*. *Applied and Environmental Microbiology*.

[98] Jahn M., Haderlein S., Meckenstock R. (2005). A novel mechanism of electron transfer from iron-reducing microorganisms to solid iron phases. *Geophysical Research Abstracts*.

[99] Roden E. E., Lovley D. R. (1993). Dissimilatory Fe(III) reduction by the marine microorganism *Desulfuromonas acetoxidans*. *Applied and Environmental Microbiology*.

[100] Kashefi K., Lovley D. R. (2000). Reduction of Fe(III), Mn(IV), and toxic metals at 100°C by *Pyrobaculum islandicum*. *Applied and Environmental Microbiology*.

[101] Kieft T. L., Fredrickson J. K., Onstott T. C. (1999). Dissimilatory reduction of Fe(III) and other electron acceptors by a thermus isolate. *Applied and Environmental Microbiology*.

[102] Liu S. V., Zhou J., Zhang C., Cole D. R., Gajdarziska-Josifovska M., Phelps T. J. (1997). Thermophilic Fe(III)-reducing bacteria from the deep subsurface: the evolutionary implications. *Science*.

[103] Zhang C., Liu S., Logan J., Mazumder R., Phelps T. J. (1996). Enhancement of Fe(III), Co(III), and Cr(VI) reduction at elevated temperatures and by a thermophilic bacterium. *Applied Biochemistry and Biotechnology A*.

[104] Ahmad A., Senapati S., Khan M. I., Kumar R., Sastry M. (2003). Extracellular biosynthesis of monodisperse gold nanoparticles by a novel extremophilic actinomycete, *Thermomonospora* sp.. *Langmuir*.

[105] Zhang C., Vali H., Romaner C. S., Phelps T. J., Liu S. V. (1998). Formation of single-domain magnetite by a thermophilic bacterium. *The American Mineralogist*.

[106] Laverman A. M., Blum J. S., Schaefer J. K., Phillips E. J. P., Lovley D. R., Oremland R. S. (1995). Growth of strain SES-3 with arsenate and other diverse electron acceptors. *Applied and Environmental Microbiology*.

[107] Lovley D., Lovley D. (2000). Fe (III) and Mn (IV) reduction. *Environmental Microbe-Metal Interactions*.

[108] Oremland R., Frankenberger W. T. J., Benson S. (1994). Biogeochemical transformations of selenium in anoxic environments. *Selenium in the Environment*.

[109] Thorek D. L. J., Chen A. K., Czupryna J., Tsourkas A. (2006). Superparamagnetic iron oxide nanoparticle probes for molecular imaging. *Annals of Biomedical Engineering*.

[110] Rogers W. J., Basu P. (2005). Factors regulating macrophage endocytosis of nanoparticles: implications for targeted magnetic resonance plaque imaging. *Atherosclerosis*.

[111] Moroz P., Jones S. K., Gray B. N. (2002). Tumor response to arterial embolization hyperthermia and direct injection hyperthermia in a rabbit liver tumor model. *Journal of Surgical Oncology*.

[112] Moroz P., Jones S. K., Gray B. N. (2002). Magnetically mediated hyperthermia: current status and future directions. *International Journal of Hyperthermia*.

[113] Love L. J., Jansen J. F., McKnight T. E. (2005). Ferrofluid field induced flow for microfluidic applications. *IEEE/ASME Transactions on Mechatronics*.

[114] Love L. J., Jansen J. F., McKnight T. E., Roh Y., Phelps T. J. (2004). A magnetocaloric pump for microfluidic applications. *IEEE Transactions on Nanobioscience*.

[115] Lloyd J. R., Yong P., Macaskie L. E. (1998). Enzymatic recovery of elemental palladium by using sulfate-reducing bacteria. *Applied and Environmental Microbiology*.

[116] de Windt W., Aelterman P., Verstraete W. (2005). Bioreductive deposition of palladium (0) nanoparticles on *Shewanella oneidensis* with catalytic activity towards reductive dechlorination of polychlorinated biphenyls. *Environmental Microbiology*.

[117] Yong P., Rowson N. A., Farr J. P. G., Harris I. R., Macaskie L. (2002). Bioaccumulation of palladium by *Desulfovibrio desulfuricans*. *Journal of Chemical Technology and Biotechnology*.

[118] Lengke M. F., Fleet M. E., Southam G. (2006). Synthesis of platinum nanoparticles by reaction of filamentous cyanobacteria with platinum(IV)-chloride complex. *Langmuir*.

[119] Narayanan K. B., Sakthivel N. (2010). Biological synthesis of metal nanoparticles by microbes. *Advances in Colloid and Interface Science*.

[120] Hunter W. J., Manter D. K. (2008). Bio-reduction of selenite to elemental red selenium by *Tetrathiobacter kashmirensis*. *Current Microbiology*.

[121] Yadav V., Sharma N., Prakash R., Raina K. K., Bharadwaj L. M., Prakash N. T. (2008). Generation of selenium containing nano-structures by soil bacterium, *Pseudomonas aeruginosa*. *Biotechnology*.

[122] Oremland R. S., Herbel M. J., Blum J. S. (2004). Structural and spectral features of selenium nanospheres produced by Se-respiring bacteria. *Applied and Environmental Microbiology*.

[123] Dwivedi S., AlKhedhairy A. A., Ahamed M., Musarrat J. (2013). Biomimetic synthesis of selenium nanospheres by bacterial strain JS-11 and its role as a biosensor for nanotoxicity assessment: a novel se-bioassay. *PLoS ONE*.

[124] Baesman S. M., Bullen T. D., Dewald J. (2007). Formation of tellurium nanocrystals during anaerobic growth of bacteria that use Te oxyanions as respiratory electron acceptors. *Applied and Environmental Microbiology*.

[125] Zare B., Faramarzi M. A., Sepehrizadeh Z., Shakibaie M., Rezaie S., Shahverdi A. R. (2012). Biosynthesis and recovery of rod-shaped tellurium nanoparticles and their bactericidal activities. *Materials Research Bulletin*.

[126] Jayaseelan C., Rahuman A. A., Kirthi A. V. (2012). Novel microbial route to synthesize ZnO nanoparticles using Aeromonas hydrophila and their activity against pathogenic bacteria and fungi. *Spectrochimica Acta A: Molecular and Biomolecular Spectroscopy*.

[127] Babitha S., Korrapati P. S. (2013). Biosynthesis of titanium dioxide nanoparticles using a probiotic from coal fly ash effluent. *Materials Research Bulletin*.

[128] Vishnu Kirthi A., Abdul Rahuman A., Rajakumar G. (2011). Biosynthesis of titanium dioxide nanoparticles using bacterium *Bacillus subtilis*. *Materials Letters*.

[129] Dameron C. T., Reese R. N., Mehra R. K. (1989). Biosynthesis of cadmium sulphide quantum semiconductor crystallites. *Nature*.

[130] Williams P., Keshavarz-Moore E., Dunnill P. (1996). Production of cadmium sulphide microcrystallites in batch cultivation by *Schizosaccharomyces pombe*. *Journal of Biotechnology*.

[131] Chan W. C. W., Maxwell D. J., Gao X., Bailey R. E., Han M., Nie S. (2002). Luminescent quantum dots for multiplexed biological detection and imaging. *Current Opinion in Biotechnology*.

[132] Barondeau D. P., Lindahl P. A. (1997). Methylation of carbon monoxide dehydrogenase from *Clostridium thermoaceticum* and mechanism of acetyl coenzyme A synthesis. *Journal of the American Chemical Society*.

[133] Bai H., Zhang Z. (2009). Microbial synthesis of semiconductor lead sulfide nanoparticles using immobilized Rhodobacter sphaeroides. *Materials Letters*.

[134] Beveridge J. T., Hughes M. N., Lee H. (1997). Metal-microbe interactions: contemporary approaches. *Advances in Microbial Physiology*.

[135] Rouch D. A., Lee B. T. O., Morby A. P. (1995). Understanding cellular responses to toxic agents: A model for mechanism-choice in bacterial metal resistance. *Journal of Industrial Microbiology*.

[136] Mohanpuria P., Rana N. K., Yadav S. K. (2008). Biosynthesis of nanoparticles: technological concepts and future applications. *Journal of Nanoparticle Research*.

[137] Angell P. (1999). Understanding microbially influenced corrosion as biofilm-mediated changes in surface chemistry. *Current Opinion in Biotechnology*.

[138] Zierenberg R. A., Schiffman P. (1990). Microbial control of silver mineralization at a sea-floor hydrothermal site on the northern Gorda Ridge. *Nature*.

[139] Harvey P. I., Crundwell F. K. (1997). Growth of *Thiobacillus ferrooxidans*: a novel experimental design for batch growth and bacterial leaching studies. *Applied and Environmental Microbiology*.

[140] Stephen J. R., Macnaughton S. J. (1999). Developments in terrestrial bacterial remediation of metals. *Current Opinion in Biotechnology*.

[141] Kang S. H., Bozhilov K. N., Myung N. V., Mulchandani A., Chen W. (2008). Microbial synthesis of CdS nanocrystals in genetically engineered *E. coli*. *Angewandte Chemie—International Edition*.

[142] Chen Y., Tuan H., Tien C., Lo W., Liang H., Hu Y. (2009). Augmented biosynthesis of cadmium sulfide nanoparticles by genetically engineered *Escherichia coli*. *Biotechnology Progress*.

[143] Kalishwaralal K., Gopalram S., Vaidyanathan R., Deepak V., Pandian S. R. K., Gurunathan S. (2010). Optimization of *α*-amylase production for the green synthesis of gold nanoparticles. *Colloids and Surfaces B: Biointerfaces*.

[144] Park T. J., Lee S. Y., Heo N. S., Seo T. S. (2010). In vivo synthesis of diverse metal nanoparticles by recombinant *Escherichia coli*. *Angewandte Chemie—International Edition*.

[145] Deplanche K., Caldelari I., Mikheenko I. P., Sargent F., Macaskie L. E. (2010). Involvement of hydrogenases in the formation of highly catalytic Pd(0) nanoparticles by bioreduction of Pd(II) using *Escherichia coli* mutant strains. *Microbiology*.

[146] Mikheenko I. P., Rousset M., Dementin S., Macaskie L. E. (2008). Bioaccumulation of palladium by *Desulfovibrio fructosivorans* wild-type and hydrogenase-deficient strains. *Applied and Environmental Microbiology*.

[147] Lengke M. F., Southam G. (2005). The effect of thiosulfate-oxidizing bacteria on the stability of the gold-thiosulfate complex. *Geochimica et Cosmochimica Acta*.

[148] Lengke M., Southam G. (2006). Bioaccumulation of gold by sulfate-reducing bacteria cultured in the presence of gold(I)-thiosulfate complex. *Geochimica et Cosmochimica Acta*.

[149] Lengke M. F., Southam G. (2007). The deposition of elemental gold from gold(I)-thiosulfate complexes mediated by sulfate-reducing bacterial conditions. *Economic Geology*.

[150] Woolfolk C. A., Whiteley H. R. (1962). Reduction of inorganic compounds with molecular hydrogen by *Micrococcus lactilyticus*. I. Stoichiometry with compounds of arsenic, selenium, tellurium, transition and other elements.. *Journal of bacteriology*.

[151] Yanke L. J., Bryant R. D., Laishley E. J. (1995). Hydrogenase I of *Clostridium pasteurianum* functions as a novel selenite reductase. *Anaerobe*.

[152] Michel C., Brugna M., Aubert C., Bernadac A., Bruschi M. (2001). Enzymatic reduction of chromate: comparative studies using sulfate-reducing bacteria: key role of polyheme cytochromes c and hydrogenases. *Applied Microbiology and Biotechnology*.

[153] Zadvorny O. A., Zorin N. A., Gogotov I. N. (2006). Transformation of metals and metal ions by hydrogenases from phototrophic bacteria. *Archives of Microbiology*.

[154] Matsunaga T., Takeyama H. (1998). Biomagnetic nanoparticle formation and application. *Supramolecular Science*.

[155] Schüler D. (1999). Formation of magnetosomes in magnetotactic bacteria. *Journal of Molecular Microbiology and Biotechnology*.

[156] Grünberg K., Wawer C., Tebo B. M., Schüler D. (2001). A large gene cluster encoding several magnetosome proteins is conserved in different species of magnetotactic bacteria. *Applied and Environmental Microbiology*.

[157] Arakaki A., Nakazawa H., Nemoto M., Mori T., Matsunaga T. (2008). Formation of magnetite by bacteria and its application. *Journal of the Royal Society Interface*.

[158] Moisescu C., Bonneville S., Tobler D., Ardelean I., Benning L. G. (2008). Controlled biomineralization of magnetite (Fe_3_O_4_) by *Magnetospirillum gryphiswaldense*. *Mineralogical Magazine*.

[159] Riddin T. L., Govender Y., Gericke M., Whiteley C. G. (2009). Two different hydrogenase enzymes from sulphate-reducing bacteria are responsible for the bioreductive mechanism of platinum into nanoparticles. *Enzyme and Microbial Technology*.

[160] Moreau J. W., Weber P. K., Martin M. C., Gilbert B., Hutcheon I. D., Banfield J. F. (2007). Extracellular proteins limit the dispersal of biogenic nanoparticles. *Science*.

[161] Amemiya Y., Arakaki A., Staniland S. S., Tanaka T., Matsunaga T. (2007). Controlled formation of magnetite crystal by partial oxidation of ferrous hydroxide in the presence of recombinant magnetotactic bacterial protein Mms6. *Biomaterials*.

[162] Prozorov T., Mallapragada S. K., Narasimhan B. (2007). Protein-mediated synthesis of uniform superparamagnetic magnetite nanocrystals. *Advanced Functional Materials*.

[163] Prozorov T., Palo P., Wang L. (2007). Cobalt ferrite nanocrystals: out-performing magnetotactic bacteria. *ACS Nano*.

[164] Mokhtari N., Daneshpajouh S., Seyedbagheri S. (2009). Biological synthesis of very small silver nanoparticles by culture supernatant of *Klebsiella pneumonia*: the effects of visible-light irradiation and the liquid mixing process. *Materials Research Bulletin*.

[165] Iravani S., Korbekandi H., Mirmohammadi S. V., Zolfaghari B. (2014). Synthesis of silver nanoparticles: chemical, physical, and biological methods. *Research in Pharmaceutical Sciences*.

[166] Shankar S. S., Rai A., Ahmad A., Sastry M. (2004). Rapid synthesis of Au, Ag, and bimetallic Au core-Ag shell nanoparticles using Neem (*Azadirachta indica*) leaf broth. *Journal of Colloid and Interface Science*.

